# The experimental study of mir‐99a‐5p negative regulation of TLR8 receptor mediated‐mediated innate immune response in rabbit knee cartilage injury

**DOI:** 10.1002/iid3.1211

**Published:** 2024-04-11

**Authors:** Jiebin Zhang, Ke Zheng, Yichao Wu, Shengting Zhang, Ao Guo, Cong Sui

**Affiliations:** ^1^ Provincial Second Clinical College of Anhui Medical University Hefei Anhui China; ^2^ Department of Orthopaedics Anhui No. 2 Provincial People's Hosipital Hefei Anhui China; ^3^ Department of Orthopaedics The First Afffliated Hospital of Anhui Medical University Hefei Anhui China

**Keywords:** articular cartilage injury, innate immune response, Mir‐99a‐5p, PI3K‐AKT, TLR8

## Abstract

**Background:**

Traumatic cartilage injury is an important cause of osteoarthritis (OA) and limb disability, and toll‐like receptors (TLRs) mediated innate immune response has been confirmed to play a crucial role in cartilage injury. In the previous study, we found that the activation of TLR8 molecules in injured articular cartilage was more obvious than other TLRs by establishing an animal model of knee impact injury in rabbits, and the changes of TLR8 molecules could significantly affect the process of articular cartilage injury and repair.

**Objective:**

To verify how mir‐99a‐5p regulates TLR8 receptor mediated innate immune response to treat traumatic cartilage injury.

**Methods:**

The impact of a heavy object on the medial condyle of the rabbit's knee joint caused damage to the medial condylar cartilage. Through pathological and imaging analysis, it was demonstrated whether the establishment of an animal model of traumatic cartilage injury was successful. Establishing a cell model by virus transfection of chondrocytes to demonstrate the role of TLR8 in the innate immune response to impact cartilage injury. Through transcriptome sequencing, potential targets of TLR8, mir‐99a‐5p, were predicted, and basic experiments were conducted to demonstrate how they interact with innate immune responses to impact cartilage damage.

**Results:**

TLR8 is a receptor protein of the immune system, which is widely expressed in immune cells. In our study, we found that TLR8 expression is localized in lysosomes and endosomes. Mir‐99a‐5p can negatively regulate TLR8 to activate PI3K‐AKT molecular pathway and aggravate cartilage damage. Inhibiting TLR8 expression can effectively reduce the incidence of articular cartilage damage.

**Conclusion:**

Based on the results from this study, mir‐99a‐5p may be an effective molecular marker for predicting traumatic cartilage injury and targeting TLR8 is a novel and promising approach for the prevention or early treatment of cartilage damage.

## Graphical abstract

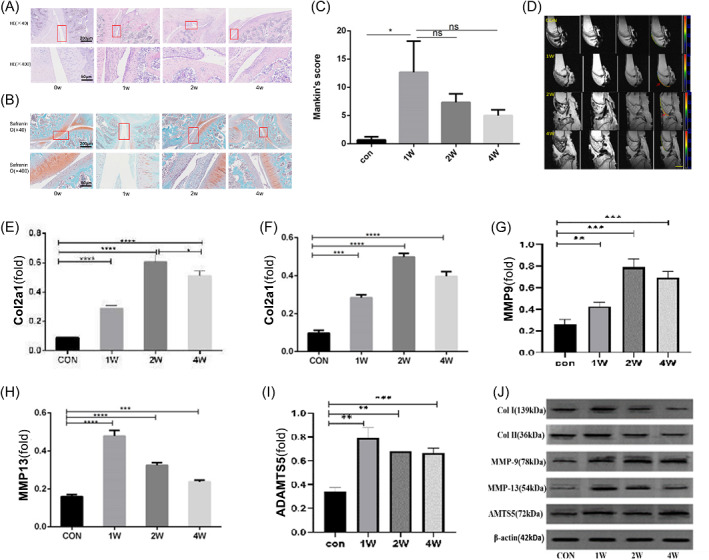

## 1 INTRODUCTION

Articular cartilage injury is a disease that widely involves people of all ages and has serious consequences. Its main causes include trauma, infection, inflammation, degeneration, congenital, and metabolic diseases. In recent years, with the expansion of injury‐causing energy spectrum and the enhancement of population aging trend, the incidence of traumatic articular cartilage injury has been increasing year by year. According to statistics, the annual number of cartilage injury cases in China is 65 million cases, and the incidence of injury in specific populations such as athletes can reach 22%−50%.^1^ The early symptoms of traumatic cartilage injury are hidden. Only 30%−40% of the patients have clinical symptoms and seek medical advice. Pathological specimens are not easy to obtain. This leads to insufficient clinical diagnosis of traumatic cartilage injury, which is an important reason why the incidence of osteoarthritis (OA) increases year by year due to cartilage degeneration in the later period.^2^

There are many methods for experimental animal models of articular cartilage injury, such as mechanical immobilization, adding local immobilization with papuloprotease, using collagenase, cutting off the medial and lateral collateral ligaments, cutting off the anterior and posterior cruciate ligaments. The medial semilunar plate was excised, and the patellar bone was excised to produce segmental instability.^3^ The human acute stress cartilage injury is often caused by collision, such as the collision between bones caused by falling from a high place, the contact collision between joints and external hard objects. The more similar the injury mechanism of animal model of cartilage injury to that of human, the more valuable the treatment and related research will be. Some scholars used the impact method to establish an animal model of knee cartilage injury in rabbits, which is simple to operate, basically similar to the pathological process of human articular cartilage injury, and has good comparability.^4^ It can simultaneously obtain normal knee joint synovial fluid samples of the same experimental animal, and is a good animal experimental model. New berry used vertical impact frames with different weights (1.33, 0.43 kg) to fall from different heights (46, 20 cm) to close the medial femoral condyle of rabbits, causing different degrees of damage to femoral articular cartilage.^5^ They found that cartilage damage was very similar to clinical pathology, which could be used as an animal model of articular cartilage damage. Mastbergen established the dog cartilage injury model by performing scratches on the cartilage surface without damaging the subchondral bone.^6^ According to the research methods of relevant literature, we improved and made a rabbit model of articular cartilage injury caused by impact of knee joint, and carried out relevant pathological staining and MRI examination to confirm that the model was successful.

Toll‐like receptors (TLRs) family is an important sensor for detecting heterogeneous pathogens and endogenous danger signals in innate immune response, and is involved in a variety of physiological processes.^7^ So far, 13 kinds of TLRs have been detected in humans and mice, of which TLR1−TLR9 is expressed in both humans and mice. TLR10 in mouse genome has no clear function, while TLR11−TLR13 is only found in mouse genome.^8^ These TLRs can recognize various microbial pathogens and synthetic ligands, including lipids, lipoproteins, polysaccharides, proteins, or nucleic acids with different molecular weights. Numerous studies have shown that the abnormal activation of TLR2 and TLR4 mediates the process of cartilage injury by affecting the activation of NF‐κB pathway.^9^, ^10^ Some studies have also shown that single gene mutation in the promoter region of TLR3 and elevated expression of TLR3 gene are associated with susceptibility to cartilage injury.^11^ Hoshikawa et al. found that TLR7 is the target of miR‐21, and miR‐21 in cartilage tissue of cartilage injury rat model can lead to cartilage damage aggravation and the occurrence of OA in the later stage by activating TLR7.^12^ The polymorphism of TLR9‐T‐1486C gene is related to the progressive aggravation of cartilage damage. The change of TLR9 expression caused by different alleles may lead to the aggravation of cartilage damage.^13^ We found that the expression of TLR1−TLR9 was increased in varying degrees in the animal model of knee cartilage injury induced by impact in rabbits, but the activation of TLR8 was more obvious than that of other TLRs.

TLR8 is expressed in a variety of physiological cells, such as monocytes, macrophages, DC cells, mast cells, and microglia cells. It is often considered as the natural receptor of single stranded RNA and a powerful activator of natural immune response after virus infection.^7^ After TLR8 is recognized and bound to the ligand, the TIR domain containing the adapter myeloid differentiation factor MyD88 begins to change. The binding of TLR8 and MyD88 stimulates the recruitment of interleukins (IL‐1) receptor‐associated protein kinases (IRAK) and leads to the activation of downstream mitogen‐activating proteins (MAPKs) and IκB kinase (IKK) complexes. Thus, the expression of MAPK family members phosphorylated activated transcription factor activator protein 1 (AP‐1) is increased, and the nuclear translocation of IKK complex and transcription factor (NF‐κB) is further promoted to control the expression of proinflammatory cytokines. In addition, members of the interferon (IFN) regulatory factor family of transcription factors can be activated in MyD88 dependent pathway and induce the production of type I IFN.^8^ In addition to its role in antiviral immunity, TLR8 also plays an important role in a variety of physiological diseases. For example, the activation of TLR8 in pancreatic cancer can promote the proliferation of tumor cells and enhance their drug resistance.^14^ In systemic lupus erythematosus, miR‐21 acts as a ligand to mediate the activation of STAT1 regulated by estrogen and promote the activation of TLR8, thereby promoting the expression of related inflammatory factors.^15^ Activation of TLR8 in ischemic stroke also promotes neuronal apoptosis and T cell‐mediated inflammation.^16^

Recent studies have found that miRNA molecules have a negative regulatory effect on TLRs signaling pathway. For example, let‐7i inhibits the translation process by interacting with the 3′ untranslated region (3′ UTR region) of TLR4 mRNA at the posttranscriptional level. TLR4 is negatively regulated.^17^ Bazzoni et al. found that miR‐9 activates the NF‐κB pathway by regulating the TLR2, TLR4, and TLR7/8 receptors of polymorphonuclear (PMN) and monocytes.^18^ These studies provide a theoretical basis for the understanding and intervention of innate immune‐related diseases. By sequencing and proteomic analysis, we found that miR‐99a‐5p could negatively regulate TLR8 receptor‐mediated innate immune responses in animal models of impingent cartilage injury. mir‐99a‐5p belongs to the miR‐99 family and is expressed at low levels in a variety of human malignant tumors and inflammatory diseases. Zhu et al.^19^ confirmed that miR‐99a is a suppressor gene, which can inhibit cell proliferation and induce cell apoptosis by regulating the activation of T cells. Jaiswal et al.^20^ found that miR‐99a may be involved in the regulation of TNF‐α and other inflammatory factors, leading to the activation of related inflammatory pathways. Other studies have shown that miR‐99a inhibits the invasion and migration of tumor cells by targeting NADPH oxidase 4 (NOX4)‐mediated reactive oxygen species (ROS) generation pathway.^21^ Mir‐99a regulates the formation and differentiation of rat mesenchymal stem cells by targeting bone morphogenetic protein receptor (BMPR2) gene.^22^

Phosphatidylinositol 3‐kinase (PI3Ks) protein family is involved in the regulation of cell proliferation, differentiation, apoptosis, glucose transport, and other cellular functions. Increased PI3K activity is often associated with a variety of cancers. PI3K phosphorylates the third carbon atom of the inositol ring of a membrane phospholipid (PI). The proportion of PI in cell membrane is smaller than that of phosphatidyl choline, phosphatidyl ethanolamine, and phosphatidyl serine, but the content of PI in brain cell membrane is more abundant, up to 10% of the total phosphatidyl. The result of PI3K activation is the generation of a second messenger, PIP3, at the plasma membrane, which binds to the PH domain‐containing signaling protein AKT and PDK1 (phosphoinositide dependent kinase‐1) in cells. Phosphorylation of AKT by PDK1 at Ser308 leads to AKT activation. AKT, also known as protein kinase B (PKB), is a major downstream effector of PI3K. PI3k/Akt signaling pathway is a signaling pathway mediated by enzyme‐linked receptors that can regulate life activities. It not only participates in the signal transduction of various production factors, cytokines and extracellular matrix (ECM), but also participates in promoting cell proliferation, inhibiting apoptosis, regulating tissue inflammation, and tumor growth and invasion. Recent studies have shown that PI3k/Akt signaling pathway is involved in the occurrence of osteoporosis, OA, osteosarcoma, and other orthopedic diseases,^23^, ^24^, ^25^ as well as in the regulation of the proliferation, differentiation, and apoptosis of osteoclasts and osteoblasts.

In this study, we found that the expression of TLR8 was significantly increased in the impact injury model of rabbit cartilage. miR‐99a‐5p can negatively regulate the innate immune response mediated by TLR8/PI3K/Akt. Targeting the abnormal activation of TLR8 molecules can effectively block the progress of cartilage injury. These results confirm that TLR8 has an important effect on early traumatic cartilage injury, and miR‐99a‐5p may become an important molecular marker to predict early cartilage injury.

## 2 MATERIALS AND METHODS

### 2.1 Experimental animals and cartilage damage model

New Zealand white rabbits (a total of 20 healthy adult New Zealand white rabbits with body weight in 3.0−3.5 kg, male and female unlimited) were obtained from the Experimental Animal Center of Anhui Medical University. All procedures were approved by the Anhui Medical University Laboratory Animal Ethics Committee (approval number: LLSC20210237). We used Borrelli's method to modify the animal model of impingement cartilage injury in rabbit knee joint. After adaptive feeding for 1 week, rabbits were modeled. The specific method was as follows: the supine position was taken, and the pelvis and thigh were fixed. Anesthesia with 10% chloral hydrate and hair removal at both knees. A longitudinal incision of about 3 cm was made on the medial side of one knee joint. The rabbit patella was everted, the knee joint was flexion and extension 120°, and the femoral shaft was 90°. A 1 kg impingent was used to fall down from a height of 50 cm along the guide rod and impinged on the medial femoral condyle, causing cartilage damage on the medial femoral condyle. All rabbits were sacrificed in a CO_2_ chamber at 4 weeks postsurgery and knee joints were collected for MRI imaging and immunohistochemistry staining.

### 2.2 Histological and immunohistochemical

All the joint tissues were demineralized in 10% EDTA for 6 weeks and embedded in paraffin after gradient dehydration. Series mid‐sagittal sections of 5 μm were cut parallel to the condyle. After dewaxing in xylene and hydration in gradient alcohol, H&E, Safranin O staining were carried out according to the manufacturer's protocol. Cartilage thickness was determined from H&E staining on the basis of the predecessors' methods. A modified Mankin scoring system was used for cartilage OA score.

After dewaxing and hydration, the tissue sections were microwave antigen‐retrieved in citrate solution. The sections were endogenous peroxidase activity blocked, following by serum blocking unspecific ligations. Rabbit anti‐TLR2, TLR4, TLR8 were incubated overnight at 4°C severally, then reacted with anti‐rabbit UltraSensitive S‐P kit according to manufacturer's protocol. For calculating the rate of positive cells, Image‐Pro Plus 6.0 software was used to quantify the number of positive cells and total cells on selected cartilage area. Anterior, middle, and posterior, three fields of each section were counted and averaged by two observers independently.

### 2.3 RT‐PCR

After total mRNA of all the rabbits cartilage samples was extracted using TRIzol Kit and reversely transcribed, polymerase chain reaction was manipulated to cDNA, with the SYBR Premix Ex Taq. Then, gene expression of mRNA was calculated using the cycle threshold method ($2^{-∆∆C_{t}}$). The primer sequences were as follows:

TLR1 F CTTGATCTGCACAATAACCG R GAAGATCCACCAAGGAGT

TLR2 F TGCCCTGTTCATTTTCCC R TTGCCTTAAAATCAACGTGTG

TLR3 F TTTCAAGGGCTTAGCTCA R AAACATTCTTGTCAACGG

TLR4 F ACAAGGCTTACTTTCACT R TCTACGCTCAGAACAGCA

TLR5 F TCTGATTCTCGCCTACCACA R TCAGGGAGAAGATAAATCCGT

TLR6 F CTGCACACATCATGAAGC R TATGTTCAATGATCAGCACAGA

TLR7 F ACAACGATATCTCCACCT R CATCTCGCCATAAAACGTCT

TLR8 F CTTTATTGGGGAAAAGCA R CAAATATTTAACATGAGGCACA

TLR9 F GGAAACCAACTGAAGAGCCTGA R TTGAGCTGTCGCAACTCCC

The gene of GAPDH was used as an internal control.

### 2.4 Western blot analysis

The collected and measured proteins were separated by SDS‐PAGE electrophoresis and then electro‐transferred onto a PVDF membrane. After blocking with nonfat milk, the membrane was incubated with different primary antibodies at 4°C overnight, respectively, including rabbit anti‐TLR2, TLR4, TLR8 (MDL). Then, HRP‐conjugated secondary antibody was incubated with PVDF membrane, and a chemiluminescence ECL system (Bio‐rad) was utilized to detect the immunoreactive proteins.

### 2.5 Double luciferase assay

The circRNAs were built into PSI‐CHECK2 vector, then circRNA and miRNA were cotransfected into 293T cells. Luciferase activities were measured at 48 h after transfection using the Dual‐Luciferase Reporter AssaySystem (Promega) according to the manufacturer's protocol. Renilla luciferase activity was normalized to firefly luciferase activity and expressed as a percentage of the control. All transfection experiments were performed in triplicate.

### 2.6 RNA pull‐down assay

The RNA was first marked by Pierce Magnetic RNA‐protein pull‐down Kit (Thermo Fisher Scientific), and then the RNA precipitated overnight was removed and centrifuged at 4°C for 30 min at maximum speed. Add 20 μL nuclease free‐water to each tube to dissolve RNA. Take 400 μL beads from each tube, place them on a magnetic frame, and remove the supernatant. Wash with 800 μL 1X binding & washing buffer for three times and suck out the supernatant. Add 400 μL 2X binding & washing buffer. Add 20 μL RNA and 380 μL DEPC water to make the final volume 800 μL, rotate the beads slowly at room temperature for 20 min to fully combine with RNA. Add 500 μL cell lysate to each tube. Meanwhile, add RNase inhibitor (1U/μL) to each tube. Rotate slowly at 4°C for 2 h to fully combine beads with cell lysis fluid. qPCR was performed after reverse transcription.

### 2.7 Plasmid DNA transfection and construction of overexpression vector

One day before transfection, plate 0.5−2 × 10^5^ cells in 500 μL of growth medium without antibiotics so that cells will be 90%–95% confluent at the time of transfection. Suspension cells: Just before preparing complexes, plate 4−8 × 10^5^ cells in 500 μL of growth medium without antibiotics. For each transfection sample, prepare complexes as follows: (a) Dilute DNA (pCDNA3.1‐EGFP‐T2A‐Puro) in 50 μL of Opti‐MEN Reduced Serum Medium without serum, mix gently. (b) Mix Lipofectamine 3000 gently before use, then dilute the appropriate amount in 50 μL of Opti‐MEN medium. Incubate for 5 min at room temperature. (c) After the 5 min incubation, combine the diluted DNA with diluted Lipofectamine 3000 (total volume = 100 μL). Mix gently and incubate for 20 min at room temperature. Complexes are stable for 6 h at room temperature. Add the 100 μL of complexes to each well containing cells and medium. Incubate cells at 37°C in a CO_2_ incubator for 18−48 h before testing for transgene expression. Medium may be changed after 4−6 h. Passage cells at a 1:10 (or higher dilution) into fresh growth medium 24 h after transfection. Add selective medium the following day.

### 2.8 Cell culture was transfected with SiRNA

TLR2SiRNA, TLR4SiRNA, and TLR8SiRNA were purchased from Huzhou Hippo Biotechnology Co., Ltd, the specific sequence is shown in Supporting Information S1. Six centimeters cell dishes were taken and 2 mL (1−2 × 10^5^ cells) culture medium was added into each well. The culture density reached 70%−90% at 37°C and 5% CO_2_. Dilute Lipofectamine 3000 (Invitrogen; No. 3000015) with Opti‐MEM and mix well. Dilute the carrier with Opti‐MEM, add P3000, and mix well. Diluted carrier (1:1) was added into the diluted Lipofectamine 3000 and incubated at room temperature for 5 min. Add the mixture into the culture medium, shake well and incubate at 37°C for 3 days.

### 2.9 Statistical analysis

All results were the averages of at least three independent experiments from separately treated and transfected cultures. Data were expressed as the mean ± SD. Statistical comparisons were performed using one‐way analysis of variance. *p* < .05 was considered to indicate a statistically significant difference.

## 3 RESULTS

### 3.1 In a rabbit model of impingement knee cartilage injury, we found an innate immune response mechanism at the early stage of injury

**Figure 1:**
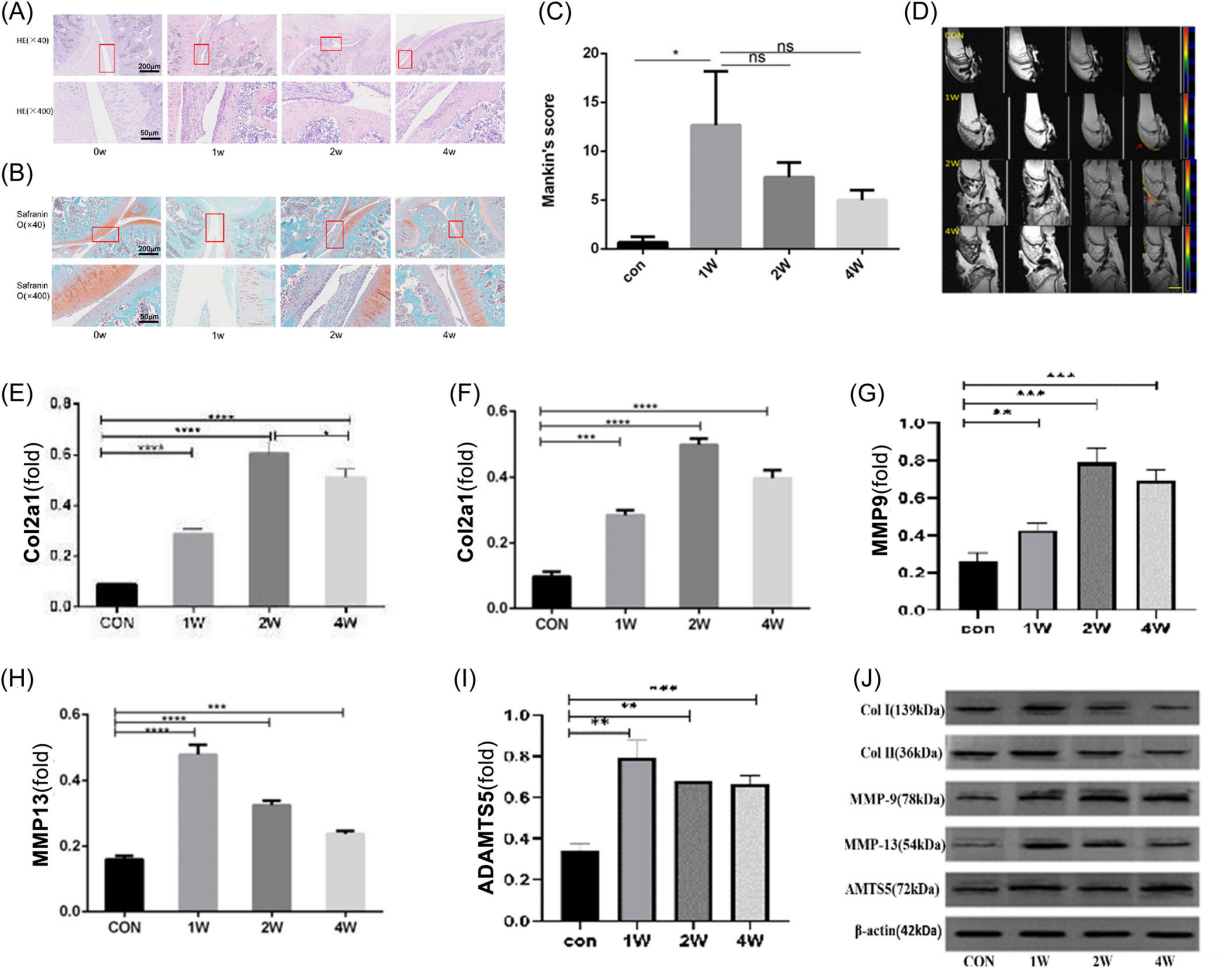
In a rabbit model of impingement knee cartilage injury, we found an innate immune response mechanism at the early stage of injury. Healthy adult New Zealand white rabbits with body weight in 3.0−3.5 kg underwent impact damage. Knee joints were harvested at 1, 2, 4 weeks after injury. *n* = 5 rabbits per group. (A) HE staining for the knee joint (sagittal view). Scale bar: 200 μm (x40), 50 μm (x400). (B) Safranin O‐Fast Green staining of the rabbit articular cartilage (0−4 weeks). Scale bar: 200 μm (x40), 50 μm (x400). (C) The modified Mankin's score used to quantify groups in the given graph, there are statistical differences between CON and 1 W group, ∗∗∗*p* < .001, ∗∗*p* < .01, ∗*p* < .05 compared with control group. (D) MRI imaging results at different time after cartilage injury in rabbits, the color area of the image is articular surface and the red arrow indicates the site of cartilage damage. (E−I) Quantification of the number of COL1A1‐positive, COL2A1‐positive, MMP9‐positive, MMP13‐positive, ADAMTS5‐positive cells in cartilage. (J) The expression levels of COL1A1, COL2A1‐, MMP9‐, MMP13, and ADAMTS5 were estimated via western blot analysis in the different weeks. ∗*p* < .05 compared with the control group. CON, control; 1−4 W, 1−4 weeks.

As described above, we applied Borrelli's method in rabbits to verify the pathological changes of impingement knee cartilage injury. According to the experimental plan, after the surgery of rabbit, we have selected 1, 2, and 4 weeks as the detection time points for research. At 1 week after injury, the articular cartilage was significantly degenerated in rabbits with impingement knee cartilage injury, as shown by HE and safranin‐O & fast green staining (Figure 1A,B) and the Mankin's scores were also significantly increased compared with those of control rabbits in 0 week (Figure 1C). All joint specimens obtained were photographed for observation before histological examination, we can observe the repair of articular cartilage injury at different time points, the cartilaginous surface of the joint after injury is repaired to varying degrees over time (Supporting Information S1: Figure 1). As time went on, the degree of cartilage damage gradually decreased, and the cartilage damage was significantly improved 4 weeks later. We also performed MRI scanning and marked imaging on the articular surface of different groups of rabbit joints, and observed that the normal articular cartilage surface was clear and smooth, with complete morphology and uniform thickness. From the result, 1 week after the injury, the cartilage injury site was obvious and clear, as it is showed by the red arrow marks. Cartilage damage was still visible 2 weeks later, however, after 4 weeks the cartilage basically returned to normal (Figure 1D).

Meanwhile, 1 week after the injury, the expression of MMP13 and MMP9, a molecule directly involved in cartilage degradation, was significantly increased compared with control rabbits of 0 week, and the expression of MMPs decreased gradually with the extension of injury time. To further quantitatively detect the expression of genes related to cartilage repair, molecular markers COL1A1, COL2A1, MMP9, MMP13, and ADAMTS‐5 were selected to be observed at different time points after cartilage injury. The results indicated that the expression levels of COL1A1, COL2A1, and MMP9 gradually increased 1 week after impaction, and reached a peak at 2 weeks after injury. Meanwhile, the expressions of MMP13 and ADAMTS‐5 peaked in the first week after injury, and then decreased gradually (Figure 1E−I). To further verify damage, we further tested the protein level of the cartilage, result of western blot (WB) analysis showed that the expressions of COL1A1, COL2A1, and MMP9 were the highest in the second week, while the expressions of mmp13 and ADAMTS5 were the highest in the third week (Figure 1H, Supporting Information S1: 2).

Based on these results, we successfully constructed an animal model of cartilage impact injury, and further verified the changes of different injury factors during the injury process through histology and molecular studies.

### 3.2 TLR8 molecule changes obviously in rabbit impingement knee cartilage injury

**Figure 2:**
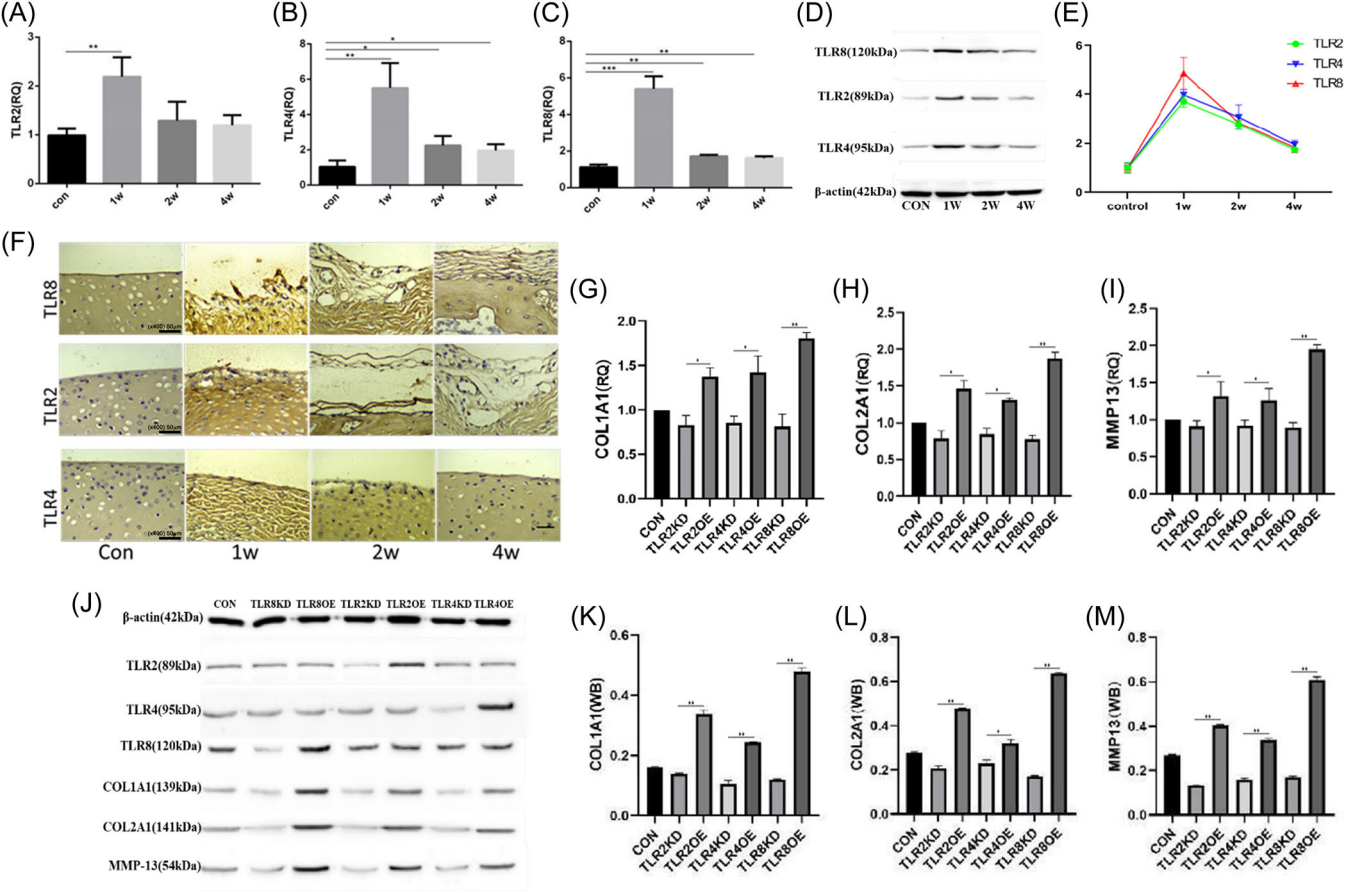
TLR8 molecule changes obviously in rabbit impingement knee cartilage injury. (A−C) The expression of TLR2, TLR4, TLR8 in each group was analyzed by q‐PCR in rabbit impingement knee cartilage injury, *p < .05. (D) The western blot analysis (WB) results of TLR2, TLR4, and TLR8 at different times, the results were consistent with q‐PCR results, and the change reached the maximum also in the first week. (E) Statistical analysis results showed that TLR8 changes were most significant in cartilage injury, *p < .05. (F) The immunohistochemical results of TLR2, TLR4, and TLR8 at different time points of injury, scale bars: 50 μm (x400). (G−I) Quantification of COL1A1, COL2A1, MMP13 expression levels with TLR2, TLR4, TLR8 knockdown (TLR2KD, TLR4KD, TLR8KD) and TLR2, TLR4, TLR8 overexpression (TLR2OE, TLR4KDOE, TLR8OE) using RT‐qPCR, ∗∗*p* < .01, ∗*p* < .05. (J−M) The expression levels of COL1A1, COL2A1, and MMP13 were estimated via WB analysis after different treatments, **p < .01, ∗*p* < .05. TLR, toll‐like receptors.

**Figure 3:**
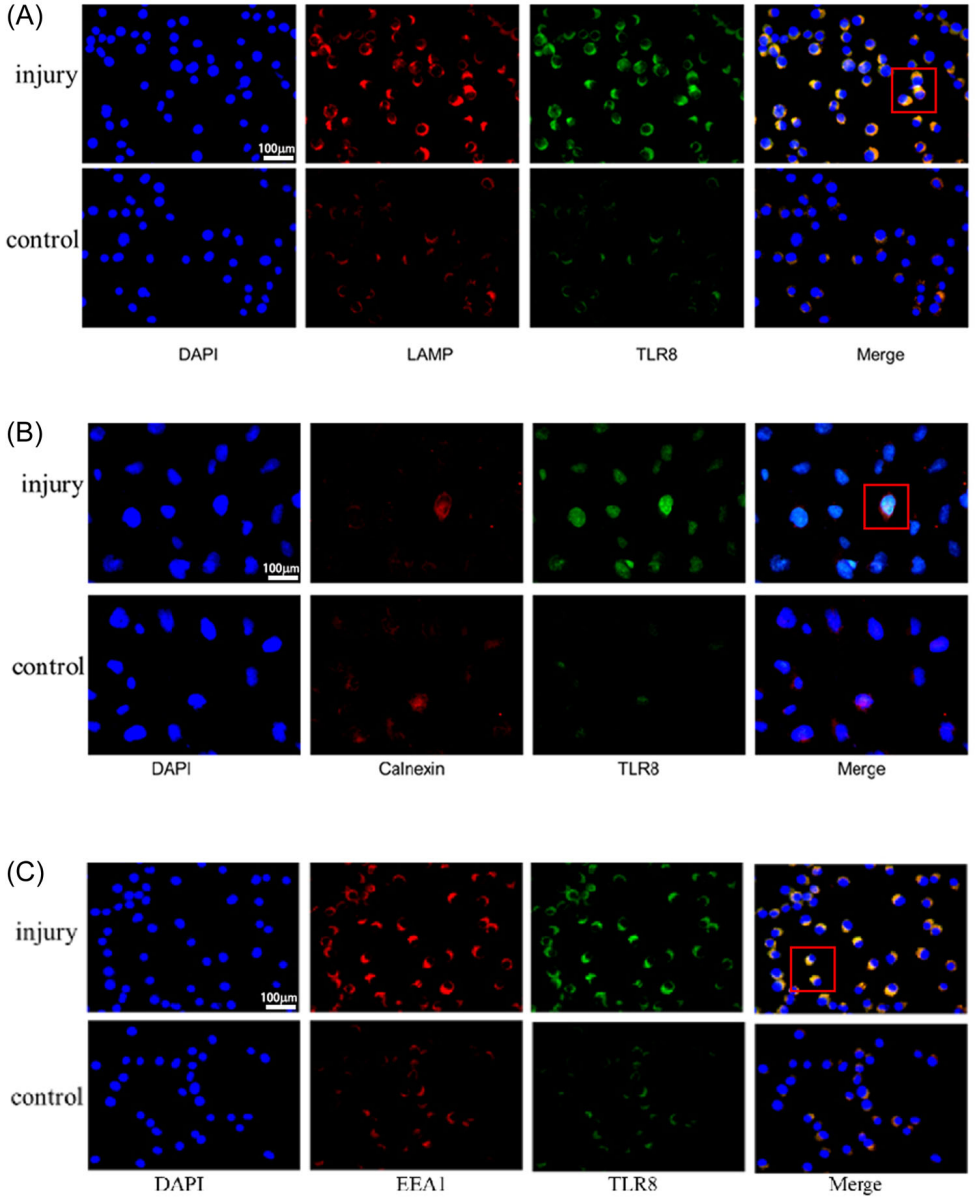
TLR8 localizes in lysosomes mediating innate immune responses during chondrocyte injury. (A) Representative images of immunofluorescence staining for LAMP (red) and TLR8 (green) in rabbit impingement knee cartilage injury in each group at 1 week. Scale bars: 100 μm. (B) Representative images of immunofluorescence staining for Calnexin (red) and TLR8 (green) in rabbit impingement knee cartilage injury in each group at 1 week. Scale bars: 100 μm. (C) Representative images of immunofluorescence staining for EEA1 (red) and TLR8 (green) in rabbit impingement knee cartilage injury in each group at 1 week. Scale bars: 100 μm. TLR, toll‐like receptors.

**Figure 4:**
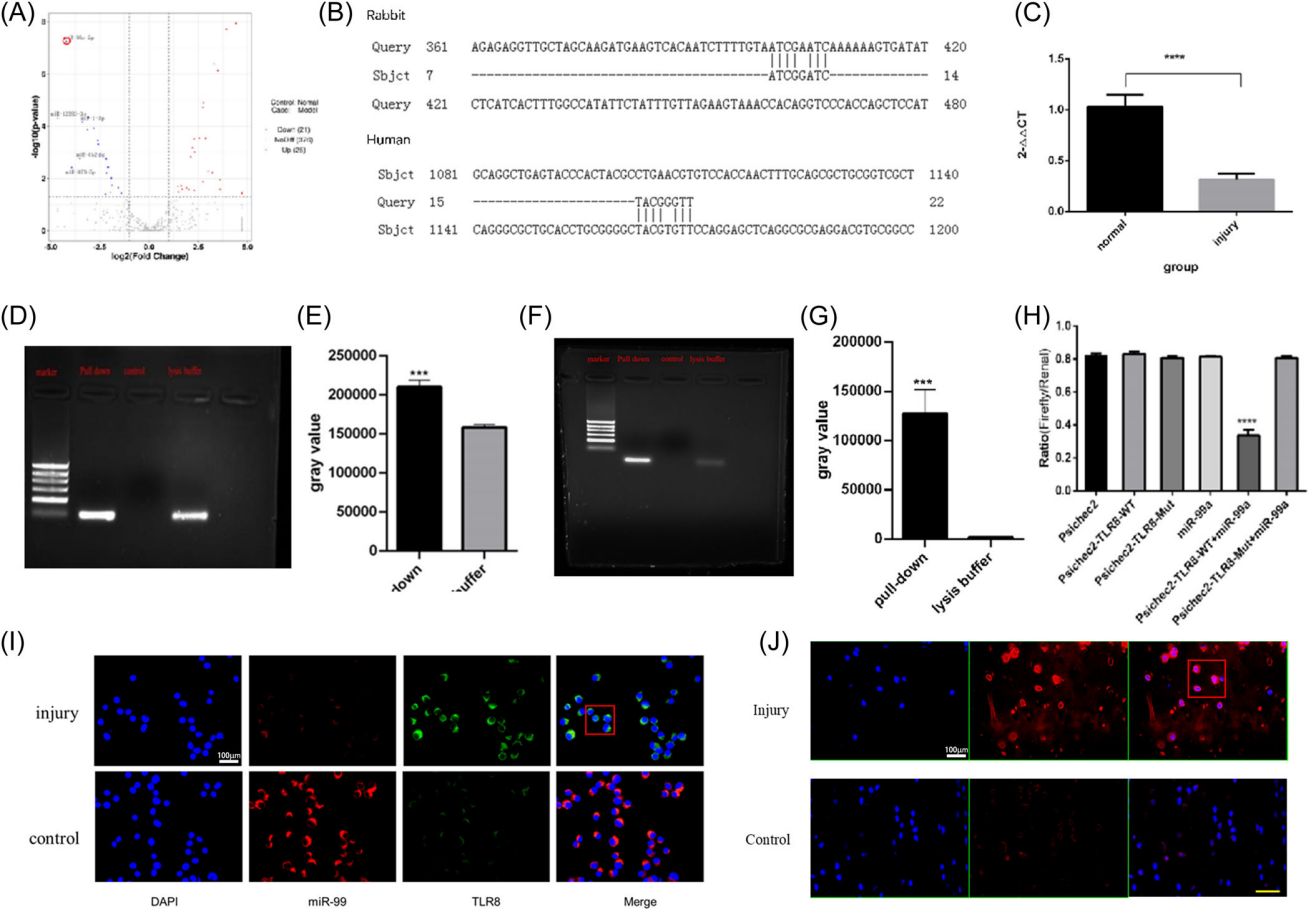
RNA sequencing of sample of impingement knee cartilage injury in rabbits and TLR8 target prediction. (A) The volcano figure of gene sequencing result, the two vertical dotted lines in the figure represent the threshold of twofold expression difference. The dotted line indicates the *p*‐value = .05. The red dots represent the upregulated genes, the blue dots represent the downregulated genes, and the gray dots represent the non‐differentially expressed genes. The abscissa is log 2 fold change, and the ordinate is –log10 (*p*‐value). (B) NCBI database search and blast comparison found that mir‐99a‐5p may bind to TLR8. (C) Clinical characteristics between the two groups, mir‐99a‐5p levels in both groups, 1.03 ± 0.04 and 0.31 ± 0.02 in group of normal and injury respectively, *p < .05. (D−G) RNA pull‐down technique verified the mutual cooperation between mir‐99a‐5p and TLR8, *p < .05. (H) The results of dual luciferase assay indicated that the binding luciferase activity of TLR8 and mir‐99a‐5p decreased, *p < .05. (I) Immunofluorescence staining for mir‐99a (red) and TLR8 (green) in the damaged cartilage of the knee. Scale bars: 100 μm. (J) Immunofluorescence staining for DAPI (blue) and NF‐κB (red) in the damaged cartilage of the knee. Scale bars: 100 μm. TLR, toll‐like receptors.

TLRs receptors are important pattern recognition receptors in innate immune response. To date, a total of 13 TLRs have been detected in humans and mice, among which TLR1−TLR10 is expressed in humans. The function of TLR10 in the human body is unclear, therefore, we detected the expression level of TLR1−TLR9 1 week after knee cartilage injury in rabbits (Supporting Information S1: Figure 3). The results showed TLR8, TLR4, TLR2 expression was significantly changed during cartilage injury, and TLR8 expression changes were significantly stronger than the others (Figure 2A−C,E). WB analysis of the injured cartilage samples showed that TLR8 was more obvious than TLR2 and TLR4 at different time points of injury (Figure 2D). Immunohistochemical staining indicated that the number of TLR8 positive cells changed significantly at different time points compared with the number of TLR2 and TLR4 positive cells (Figure 2F). We further developed a model of impact‐type chondrocyte injury in vitro to overexpress or inhibit the expression of TLRs to verify its effect on cartilage injury. Normal cartilage cells of rabbit knee joint were extracted and cocultured with the damaged joint fluid of rabbit knee joint cavity after 1 week of cartilage injury. After 24 h of coculture, TLR2, TLR4, and TLR8 genes in chondrocytes were overexpressed and inhibited to observe whether they had repair effects on injured chondrocytes. We build virus plasmid (Pcdna3.1‐EGFP‐T2A‐Puro, Supporting Information S1: Figure 4) which contain CDS region of the gene transfected into cells to overexpress the gene of TLR2, TLR4, and TLR8, at the same time use si‐RNA transfected into cells for downregulate the expressions of TLR2, TLR4, and TLR8. The overexpression and inhibition of TLR8 had the most significant effect on the expression of MMP13, COL1A1, and COL2A1 mRNA (Figure 2G−I). Meanwhlie, the overexpression and inhibition of TLR8 also affect the expression of MMP13, COL1A1, and COL2A1 at the protein level (Figure 2J−M). From the above, we conclude that TLR8 is the main pattern recognition receptor in the innate immune response to impingement cartilage injury.

### 3.3 TLR8 localizes in lysosomes mediating innate immune responses during chondrocyte injury

Previous studies have shown that TLRs may have different subcellular distribution in different cells. TLR8 in the immune system is mainly located in the cytoplasmic endosomes, while its location in cartilage organelles remains unclear. To determine the expression distribution of TLR8 in chondrocytes, the colabeling of TLR8 with lysosomal marker LAMP, endoplasmic reticulum marker Calnexin, and endosomal marker EEA1 was detected by the double‐target immunofluorescence method, respectively. The results showed that TLR8 was mainly colabeled with the lysosomal marker LAMP (Figure 3A) and the endosomal marker EEA1 (Figure 3C) in rabbit chondrocytes with impact knee joint injury, but less colabeled with the endoplasmic reticulum marker calnexin (Figure 3B). Therefore, we concluded that TLR8 was mainly expressed in lysosomes and endosomes, and there was no intracellular metastasis of TLR8 after chondrocyte injury.

### 3.4 RNA sequencing of sample of impingement knee cartilage injury in rabbits and TLR8 target prediction

**Figure 5:**
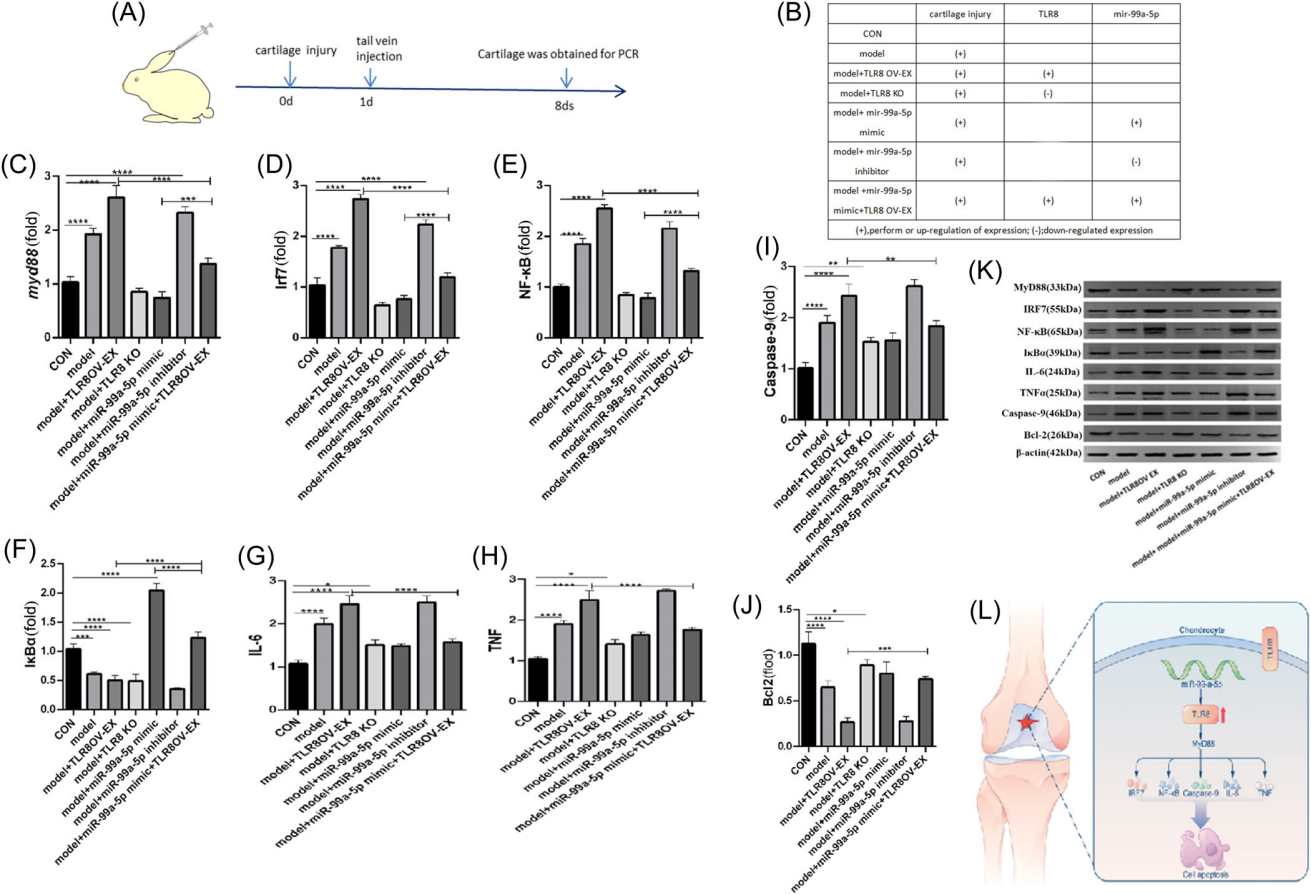
miR‐99a‐5p negatively regulates TLR8 in the occurrence of impingement cartilage injury of rabbit knee joint. (A) Different vectors were injected into the auricular vein of the cartilage‐damaged rabbits. (B) Specific grouping and intervention measures of animal experimental model. (C−F) The expression of toll‐like receptor signaling pathway related genes MyD88 (C), IRF7 (D), NF‐κB (E), and IκBα (F). (G−J) Detection of cytokines related to cell inflammatory apoptosis: IL‐6 (G), TNF (H), caspase‐9 (I), and BCL2 (J). (K) The protein expression levels of MyD88, IRF7, NF‐κB, Iκ‐Bα, IL‐6 (G), TNF, caspase‐9, and BCL2 in different groups by western blot analysis test. (L) Schematic diagram of mechanism signal illustration that mir‐99a‐5p negatively regulates the expression of TLR8 to affect the occurrence of chondrocyte innate immune response (*p < .05). CON, control group; KO, knockout; OV‐EX, overexpression; TLR, toll‐like receptors.

Related studies have reported that the TLRs receptor‐mediated innate immune responses are mostly mediated by miRNAs. It has been reported that TLR8 and its endogenous ligand miR‐21 contribute to neuropathic pain in murine DRG.^26^ There is no literature report that the congenital immune response of TLR8‐mediated cartilage injury is mediated by which miRNA species. Therefore, we performed transcriptome sequencing of specimens from the rabbit cartilage damage model. The expression level of miRNA was detected in samples of control cartilage and in cartilage 1 week after injury. DESeq (Version 1.18.0) was used to analyze the differential expression of miRNA (Figure 4A). According to the expression quantity ratio difference (| log2FoldChange | & gt; 1) and significant difference in expression (*p*‐value; .05), differential conserved mi‐RNAs were screened. The results showed that 21 miRNA downregulated and 26 mRNA upregulated, among which mir‐12093‐3p, mir‐99a‐5p, mir‐873‐5p, mir‐452‐5p, and mir‐1‐3p were the top five miRNAs with the most significantly downregulated expression, mir‐12093‐3p and mir‐133‐3p had no relevant information in the database. Therefore, mir‐99a‐5p with the highest degree of difference was selected for verification. Then through the NCBI database search and blast comparison found that mir‐99a‐5p may bind to TLR8. We can see that there is a conserved binding site in both rabbits and humans (Figure 4B). To further understand the changes of in the mir‐99a‐5p human body, the blood samples were collected from patients who diagnosed with cartilage injury and normal patients respectively, and then analyze the level of mir‐99a‐5p. Thirty‐two patients were included in each group, and their clinical data can be seen in Supporting Information S1: Table 1 (Supporting Information S1: Figure 5). The blood level of mir‐99a‐5p was 1.03 ± 0.04 and 0.31 ± 0.02 in group of normal and injury respectively, *p* < .05 (Figure 4C).

**Figure 6:**
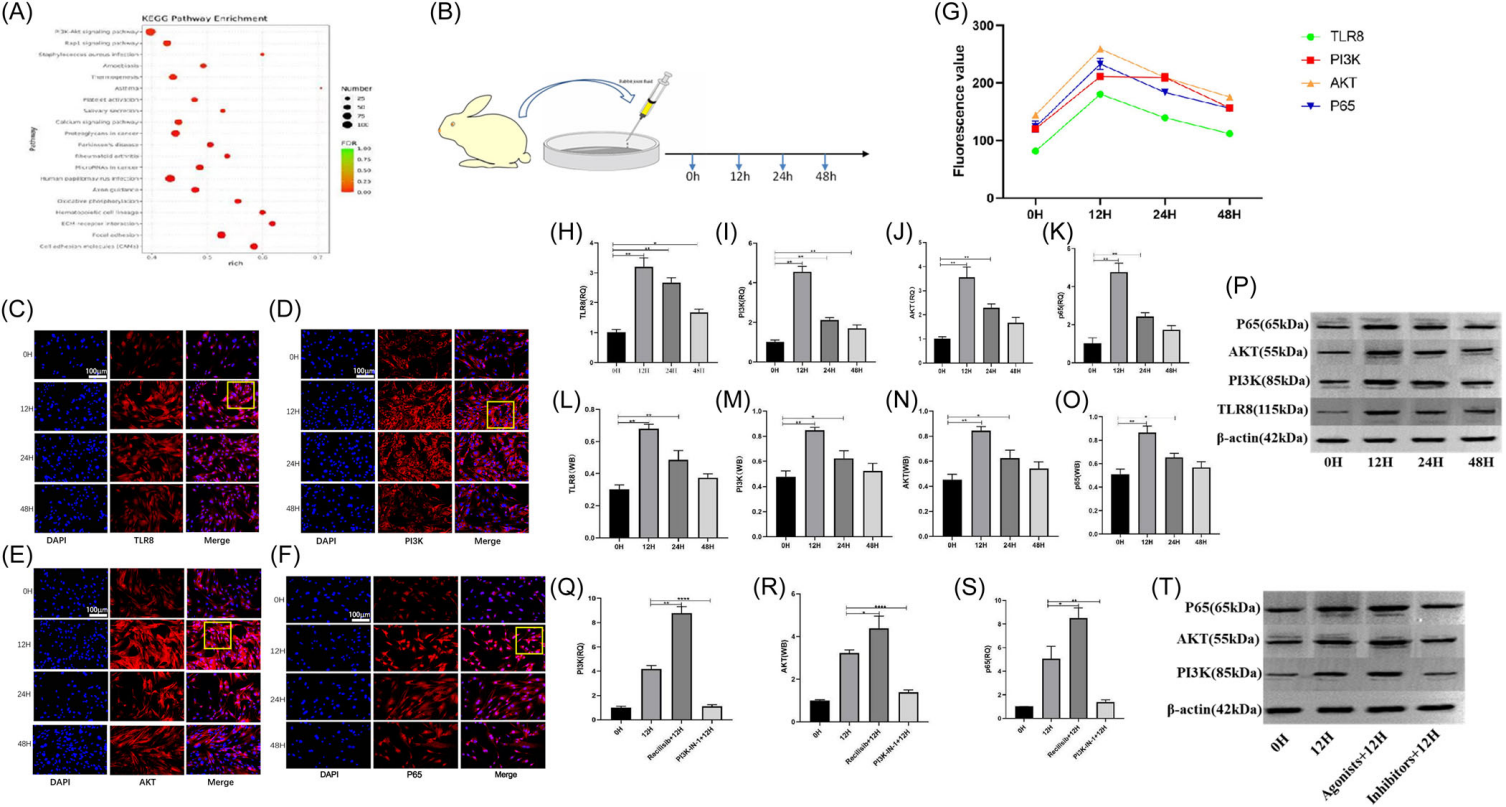
mir‐99a‐5p negatively regulates TLR8 activation of PI3K/AKT pathway to regulate knee cartilage injury in rabbits. (A) KEGG enrichment analysis that PI3K/AKT pathway is involved in TLR8‐mediated inflammatory response. (B) Schematic diagram of chondrocyte culture. (C) Immunofluorescence staining for TLR8 (red) and DAPI (blue) in the damaged cartilage of the knee. Scale bars: 100 μm. (D) Immunofluorescence staining for PI3K (red) and DAPI (blue) in the damaged cartilage of the knee. Scale bars: 100 μm. (E) Immunofluorescence staining for AKT (red) and DAPI (blue) in the damaged cartilage of the knee. Scale bars: 100 μm. (F) Immunofluorescence staining for P65 (red) and DAPI (blue) in the damaged cartilage of the knee. Scale bars: 100 μm. (G). Trend chart speculated that PI3K, P65, and AKT are synchronized with TLR8 in expression change. (H−K) PCR detection the expression levels of TLR8, PI3K, AKT, and P65 of cell culture, *p < .05. (L−P) The result of protein level of TLR8, PI3K, AKT, and P65 detected by WB，*p < .05. (Q−T) After use the PI3K inhibitor and agonist, the express level of AKT and P65 were detected by PCR (Q−S) and WB (T) respectively, the change of them is same to PI3K. PI3K, phosphatidylinositol 3‐kinase; TLR, toll‐like receptors; WB, western blot analysis.

To verify the interaction between mir‐99a‐5p and TLR8, we first using RNA pull‐down techniques find when mir‐99a‐5p as the probe, it could be seen that TLR8 in mir‐99a‐5p probe group was significantly enriched and the average *C*_t_ values was 17.93, significantly decreased compared with the cell lysis buffer (Supporting Information S1: Figure 6). After agarose gel electrophoresis, gray values of electrophoresis bands were calculated. The results showed that the amplification bands of Mir‐99a‐5p probe group were significantly stronger than cell lysates (Figure 4D,E). Then the TLR8 probe was labeled with the same method, and mir‐99a‐5P in the TLR8 probe group was significantly enriched, and the gray value of the amplified band in the TLR8 probe group was significantly stronger than that in the cell lysate (Figure 4F,G). Therefore, through the positive and negative RNA pull down experiments of mir‐99a‐5p and TLR8, it is likely that mir‐99a‐5p binds and interacts with TLR8.

To further clarify the relationship between them, we performed a double luciferase assay. The results show that the average fluorescence value ratio of Psichec2‐TLR8‐WT+miR‐99a group is 0.33, much lower than the other groups (*p* < .05) (Figure 4H), which suggested that the activity of luciferase decreased after TLR8 was combined with mir‐99a‐5p, indicating the interaction of the 3‐terminal noncoding region of mir‐99a‐5p and TLR8. At the same time, we also carried out double fluorescence on cells, found that the expression of TLR8 increased and mir‐99a‐5p decreased at the same time, and the position of the cells was similar (Figure 4I). To further illustrate the changes of related immune‐involved factors in cells after cartilage injury, we selected NF‐κB factor for detection and found that it is activated after injury and transferred to the nucleus (Figure 4J). This suggests that TLR8 activation in cartilage injury can mediate inflammatory molecular pathways.

### 3.5 miR‐99a‐5p negatively regulates TLR8 in the occurrence of impingement cartilage injury of rabbit knee joint

To gain further understanding of the downstream molecular mechanisms of cartilage damage underlying TLR8 activation, we injected mir‐99a‐5p as well as the overexpression and inhibitory vector of TLR8 in the auricular vein of model rabbits, related inflammatory properties and apoptosis‐related indicators were detected 1 week later (Figure 5A). Animal models were divided into six groups, depending on the rabbit treatment pattern, from no treatment in the control group to overexpression of mir‐99a‐5p mimic and TLR8 after cartilage injury (Figure 5B). All groups of animals underwent further testing 1 week after receiving the corresponding treatment, PCR analysis of cartilage tissue in each group showed when the TLR8 overexpression or mir‐99a‐5p decline, the MyD88 (Figure 5C), IRF7 (Figure 5D), NF‐κB (Figure 5E) also increased expression, but Iκ‐Bα is decrease when TLR8 overexpression (Figure 5F). They are accompanied by TLR8 or mir‐99a‐5p changes, suggesting them involved in the activation of the signaling pathway during cartilage injury. We further detected the apoptosis related factors of IL‐6, TNF, caspase‐9, and BCL2, and found that with the TLR8 overexpression or mir‐99a‐5p decline, IL‐6, TNF, and caspase‐9 is become higher expression (Figure 5G−I), however the BCL2 expression is opposite (Figure 5J). As above to the changes of TLR8 and mir‐99a‐5p, indicating that they may involve in various regulation and effects in cartilage injury. At the same time, WB test was used to further confirm the protein expression levels of MyD88, IRF7, NF‐κB, Iκ‐Bα, and the results were basically consistent with the PCR results (Figure 5K). Base on this study, we can infer that in the cartilage repair model, mir‐99a‐5p negatively regulates the expression of TLR8 and affect the occurrence of chondrocyte innate immune response (Figure 5L).

### 3.6 mir‐99a‐5p negatively regulates TLR8 activation of PI3K/AKT pathway to regulate knee cartilage injury in rabbits

Through the previous sequencing, many pathways were found to be involved in the immune response after cartilage injury, we can find the PI3K/AKT‐related pathway is highly correlated (Figure 6A). Based on this, we chose the PI3K/AKT related pathway as the research object to explore whether TLR8 achieves immune response through this pathway. First, the joint fluid in the rabbit trauma model (1 week) was cocultured with normal chondrocytes, and then different time points from 0 to 48 h were selected as the study nodes (Figure 6B). Then, the fluorescence values of PI3K, AKT, P65, and TLR8 were detected by immunofluorescence. According to the fluorescence map, the fluorescence values showed different changes with the change of time (Figure 6C−F). These changes suggest that this pathway is involved in the immune response to cartilage injury. Second, fluorescence values of PI3K, AKT, P65, and TLR8 was further analyzed, we can find that the value of 12 h is the highest, which are statistically different from 0 and 12 h (Supporting Information S1: Figure 7). Both PI3K, AKT, P65, and TLR8 reached the highest value at 12 h, indicating that they had synchronous changes. It is speculated that TLR8 may achieve immune response through the PI3K/AKT pathway. That is, it reaches its peak at 12 h, and then become gradual decline with the time go on (Figure 6G).

Next, the cultured cells were then further measured for PI3K, AKT, P65, and TLR8 expression levels. First, PCR detection results can be seen after 12 h of cell culture, the expression levels of PI3K, AKT, P65, and TLR8 reached the highest, and then gradually decreased, and their changing trends were basically the same (Figure 6H−K). Such results were also further confirmed by WB detection (Figure 6L−P).

To further determine whether AKT pathway is involved in immune response, PI3K inhibitor (PI3K‐IN‐1, MCE HY‐12068) and agonist (Recilisib, MCE HY‐101625) were added in cell culture, respectively. After 12 h coculture, AKT and P65 showed synchronous changes with the inhibitor or agonist, it can be reflected by PCR and WB results (Figure 6Q−T, Supporting Information S1: S8). Therefore, we boldly hypothesized that TLR8 regulates the related inflammatory response through the PI3K/AKT pathway in the early immune response after cartilage injury.

## 4 DISCUSSION

Cartilage injury is very common in clinic, which mainly includes chronic injury and acute injury. Acute injury, such as OA or rheumatoid arthritis, is mainly caused by chronic cartilage lesions. However, acute injuries are mainly caused by trauma, such as tibial plateau fracture and ankle fracture which involving in the articular surface.

Most studies focus on chronic cartilage injury, such as OA and RA. Meanwhile, many animal models of chronic cartilage injury have been established, such as DMM, ACTL, type II collagenase, and so on. Although these models were able to approximate the process of chronic cartilage injury, they were not able to reflect acute cartilage injury. Therefore, to better study the immune response process of acute cartilage injury, we chose the cartilage impingement model. It can not only approximate the process of trauma, but also mainly study the short‐term immune response after trauma.

Through the study on the impact injury model of rabbit knee joint, we found that the cartilage injury was the most severe and the Mankin's score was the highest about 1 week after the injury, and the expression changes of COL1A1, COL2A1, and MMP13 were also the largest at this time. This simultaneous change in histology and molecular. This indicates that the inflammation peaks in the first week after cartilage injury.

Many studies of articular cartilage injury have shown that there is a strong inflammatory response to joint injury. This reaction has been shown to involve synovial cells, chondrocytes, and bone cells in and around injured joints, and if left unchecked, can lead to posttraumatic OA.^27^ Cartilage cells and ECM due to trauma, degeneration of debris all can become DAMP, and then identified by TLRs receptors on the surface of the cartilage cells, inflammatory signaling pathways and activate the corresponding release decomposition factor, cause the damage of cartilage cell apoptosis, but the release of inflammatory factors can lead to the excessive release of DAMP, thus forming a vicious cycle, exacerbates inflammatory response and chondrocyte apoptosis.^28^

It is important to understand these inflammatory responses and develop successful intervention strategies to treat and ultimately prevent arthritis following joint injury. Oxidative stress is associated with the occurrence and development of OA,^29^ and studies have shown that the severity of OA can be reduced by the use of antioxidants or drugs that target ROS mechanisms in joints. Cartilage injury is accompanied by physiological processes such as chondrocyte proliferation, subchondral bone remodeling, and chronic synovitis. Production of intra‐articular proinflammatory cytokines such as ILs leads to production of ROS, such as peroxide, hydroxyl radical, and nitric oxide (NO), accompanied by downregulation of antioxidants such as superoxide dismutase, catalase (CAT), and glutathione peroxidase (GPX).^30^, ^31^ The resulting oxidative stress leads to upregulation of decomposition enzymes, degradation of the ECM, reduced matrix synthesis, joint inflammation, and chondrocyte death and senescence, thereby contributing to the overall progression of the disease.^32^ In OA, when articular cartilage is subjected to excessive mechanical load, mitochondria within chondrocytes release ROS, resulting in chondrocyte death, joint tissue inflammation, and degradation of matrix components.^32^ Clearly, oxidative stress is implicated in the progression of various phenotypes of OA, making it a therapeutic target that could impact a broad spectrum of patients. In acute injury, a large number of inflammatory mediators such as IL‐1B, IL‐6, IL‐8, TNF‐α, and NO were found in the surrounding tissues and synovial fluid, indicating that many inflammatory factors were involved in cartilage injury.^28^, ^33^

In the process of cartilage damage, DAMP, as an alarm signal, is transmitted by necrotic and damaged cells to the surrounding environment and activates immune cells. These endogenous molecules interact with cellular receptors (TLRs) to further stimulate innate immune responses that initiate signaling pathways that can trigger additional joint inflammation or initiate tissue repair.^34^ Most domestic and foreign studies have shown that the abnormal activation of TLR2 and TLR4 in OA aggravates cartilage injury by affecting the activation of NF‐κB pathway. Studies have also shown that single gene mutations in the TLR3 promoter region and increased TLR3 gene expression are associated with the susceptibility to cartilage injury.^35^

Our findings also indicated that TLR8 expression was the highest 1 week after cartilage injury, which was consistent with cartilage morphology and expression of related factors such as COL1A1 and so on. In addition, TLR1‐9 showed the greatest variation in TLR8 expression, indicating its highest participation in inflammatory immunity after cartilage injury. To better verify the involvement of TLR8 in immune response after cartilage injury, we constructed plasmids and SiRNA to overexpress or inhibit the expression of TLR2, TLR4, and TLR8, respectively; found they all changed COL1A1, COL2A1, and MMP13 to varying degrees, among which TLR8 had the greatest change. It was also found that the expression of TLR8 and CCK8 was inversely proportional, indicating that when TLR8 increased, the proliferation ability of cells was inhibited, and further experiments showed that TLR8 could promote the phagocytosis ability of cells. That results are consistent with expectations, indicating that TLR8 is involved in the immune process after cartilage injury and has an important role.

What are some of the molecules involved in the immune processes that TLR8 is involved in? First, bioinformatics analysis revealed an interesting mir‐RNA, mir‐99a‐5P, which is changed the most. Second, further research found that TLR8 was mainly expressed in the endosomes and lysosome of cells, and the expression sites of Mir‐99a‐5p were found to be highly overlapped, suggesting that the two may be highly correlated. Third, the binding between mir‐99a‐5p and TLR8 was further confirmed by RNA pull‐down experiment. Base on the research, mir‐99a‐5p may plays a negative feedback role in the TLR8‐mediated immune response.

How to evaluate the severity of the cartilage damage more effectively in clinical practice is always a problem, including the clinical symptoms of patients, such as pain and range of motion, as well as radiographic evaluation. However, combining inflammatory factors to detect and measure cartilage injury is a meaningful research direction. Through a comparative study on the content level of mir‐99a‐5p blood in human body, we find that when cartilage was damaged, the level of mir‐99a‐5p in the blood of a patient with a cartilage injury was lower than normal, and there was a statistical difference. This is an interesting phenomenon, so we speculate that when cartilage damage worsens, namely when OA occurs, due to the high expression of TLR8, the expression of mir‐99a‐5p is negatively regulated. Here we make a bold assumption that whether mir‐99a‐5p can be used as an indicator of cartilage damage in clinical practice is worth further discussion and research.

To further clarify the relationship between them, we selected rabbits as research objects and treated them with inhibition and high expression of TLR8 and mir‐99a‐5p, respectively. We detected the related factors involved in cartilage injury immunity and found that TLR8 and mir‐99a‐5p not only affected the transcription of MyD88, IRF7, Iκ‐Bα, and NF‐κB, but also participated in the process of cell apoptosis, such as IL‐6, TNF, caspase‐9, and BCL2. However, the effect of mir‐99a‐5p and TLR8 presents the opposite. By double fluorescence it not only reconfirmed the expression of TLR8 and its tight binding to mir‐99a‐5p, but also revealed that nuclear transfer of NF‐κB occurred after cartilage injury, which confirmed the effect of TLR8 on NF‐κB.

Through the above studies, we found the importance of TLR8 in cartilage injury immunity and clarified its localization in cells. The close relationship between Mir‐99a‐5p and TLR8 was found, and TLR8 was confirmed to be involved by analyzing the changes of mir‐99a‐5p. Moreover, the alteration of mir‐99a‐5p provides a research direction for cartilage injury in clinical work, and it may become a marker of cartilage injury. Of course, this study only found the changes of mir‐99a‐5p and TLR8 in cartilage injury, and we need to further clarify and study their mutual relationship and mechanism.

To further explore the specific process of TLR8 involvement in inflammation, we selected the highest correlation relevant PI3K/AKT pathway as the research object. Through coculture of rabbit articular fluid in the trauma model and normal cartilage, it could be found that, PI3K, Akt, P65, and TLR8 reached the highest value at 12 h after culture. Through their synchronous performance, we speculated that PI3K/Akt pathway was involved in TLR8‐mediated inflammatory response

This is the first study to find the change of TLR8 in cartilage injury, which confirms that tlr8 is involved in cartilage repair and plays an important role in innate immunity through two experimental animals. At the same time, an important mRNA mir‐99a‐5p was found by gene analysis, and the interaction and close correlation between mir‐99a‐5p and TLR were verified by pull‐down and other techniques, providing a clinical research direction and therapeutic choice for the treatment of cartilage injury. Of course, this study also has its limitations. First of all, we only conducted preliminary zoological studies, and further human related histological studies are needed. Second, further discussion on mechanism is needed to provide stronger support for our conclusion.

## AUTHOR CONTRIBUTIONS

Cong Sui designed this study. Jiebin Zhang and Ke Zheng completed specimen experiments. Shengting Zhang and Ao Guo performed cell culture studies. Cong Sui and Ke Zheng performed in vivo studies. Jiebin Zhang collected data. Cong Sui and Yichao Wu analyzed data. Cong Sui revised the article.

## CONFLICT OF INTEREST STATEMENT

The authors declare no conflict of interest.

## ETHICS STATEMENT

Clinical specimen experiments and animal experiments were reviewed and approved by the Ethics Committee of the First Affiliated Hospital of Anhui Medical University. The procedures used in this study adhere to the tenets of the Declaration of Helsinki.

## Supporting information

Supporting information.

## Data Availability

The data used to support this study are included within the article.
