# Supplementary material for: The experimental study of mir‐99a‐5p negative regulation of TLR8 receptor mediated‐mediated innate immune response in rabbit knee cartilage injury
Source: Immun Inflamm Dis. 2024 Apr 11;12(4):e1211. doi: 10.1002/iid3.1211 (PMC11007787; doi:10.1002/iid3.1211)
Supplement: Supplementary file 1 — Supporting information. [file IID3-12-e1211-s001.docx]

**Attached figure**


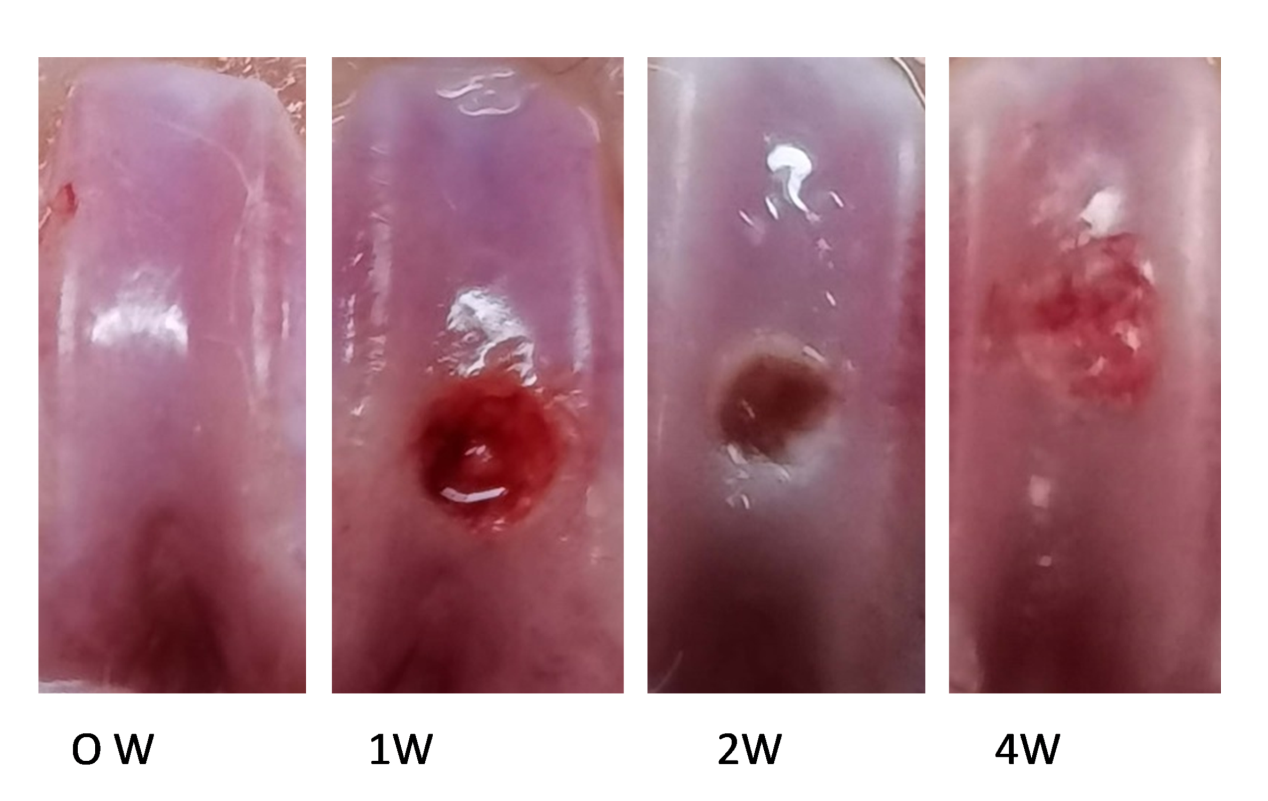


**Fig S1:** From left to right, time change 0 to 4 weeks, the gross view of impact damage the joint. The damage is most obvious at one week, and the repair of cartilage formation on the surface of the joint can be seen at four weeks


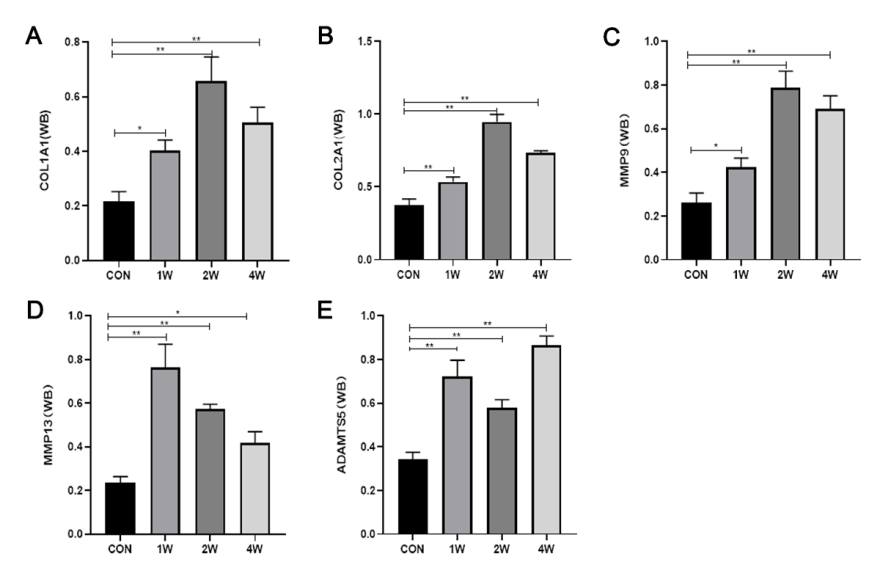


**Fig S2:** The result of western blot expression that COL1A1, COL2A1 and MMP9 were the highest in the second week, while the expressions of mmp13 and ADAMTS5 were the highest in the third weeks. *p<0.05.


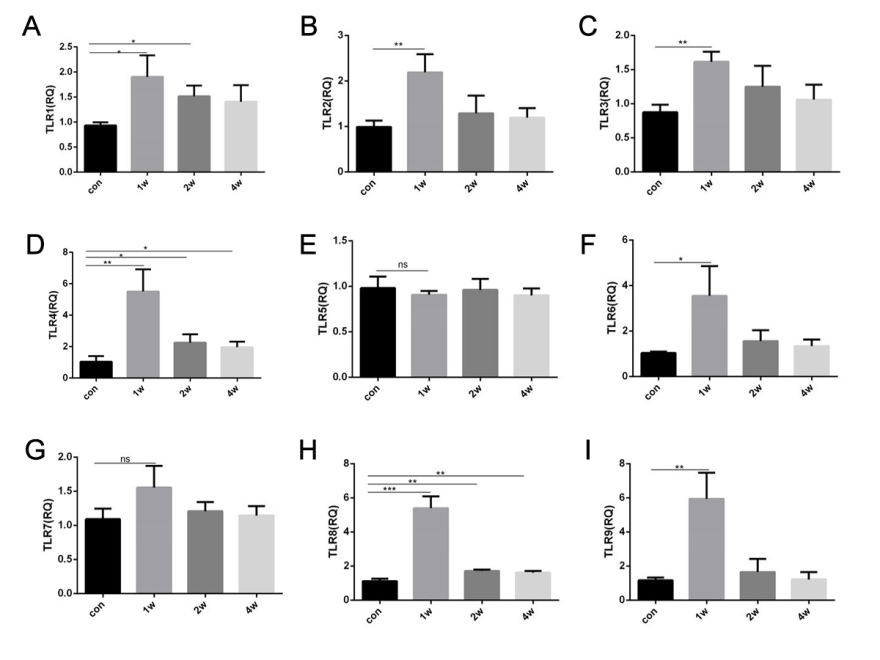


**Fig S3:** PCR and statistical analysis results of TLR1-9 expression in cartilage at different time points. *p<0.05;**ns**, no statistical difference.


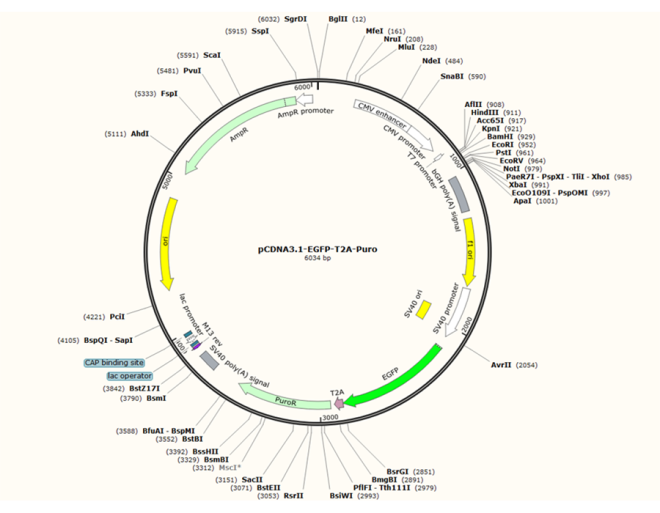


**Fig S4:** The plasmid profile of Pcdna3.1-EGFP-T2A-Puro and sequence reference points

|  | Injury(mean,SD) | Normal(mean,SD) | P |
| --- | --- | --- | --- |
| Gender (M/F) | 6/10 | 7/9 |  |
| Age(years) | 64.7 ± 3.1 | 57.7 ± 4.3 | 0.41 |

**FigS5:** Clinical characteristics between the two groups peoples, no statistical difference between the two groups,p=0.41.


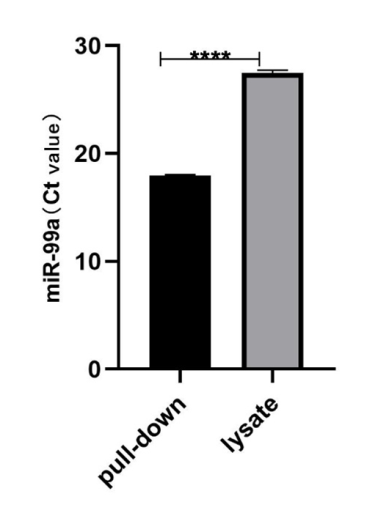


**FigS6:** Expression of TLR8 in mir-99a-5p probe group was significantly enriched and the average CT values was 17.93, significantly decreased compared with the cell lysis buffer, p<0.05.


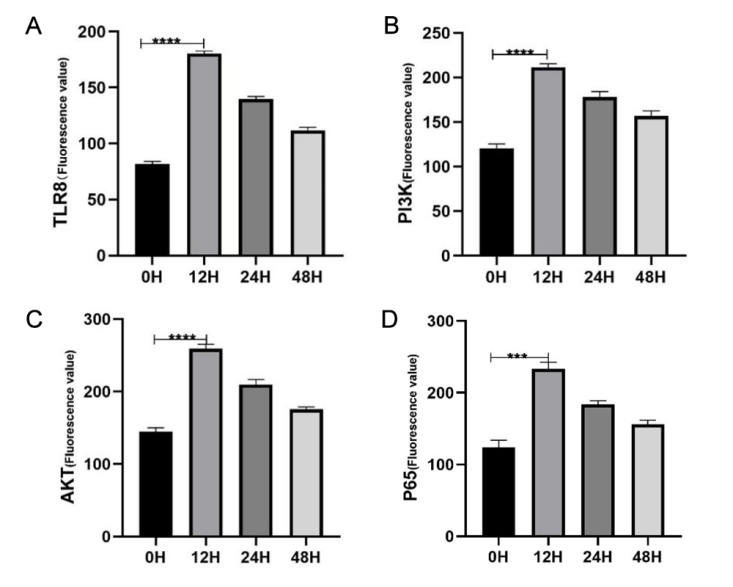


**Fig.S7:** Quantification of fluorescence values with different groups of TLR8, were compared and analyzed, and the highest value was found at 12 hours with statistical significance,*P < 0.05.


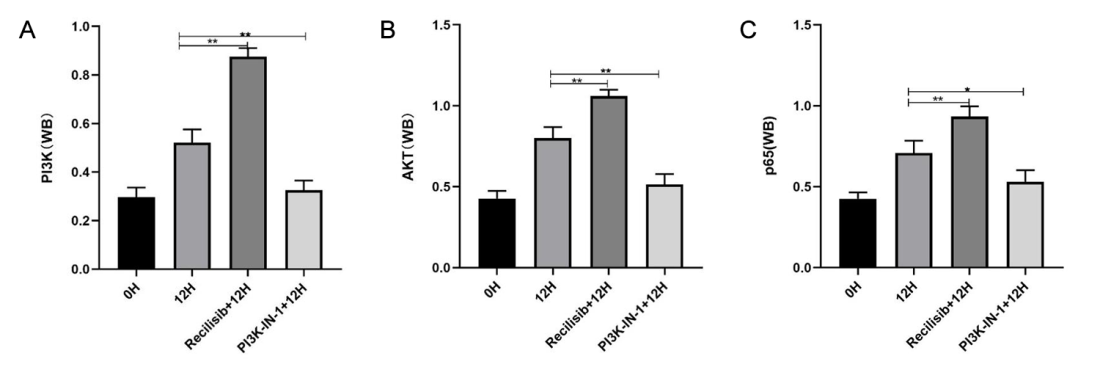


**Fig.S8:** Western blotting results showed that when PI3K was changed, AKT and P65 also had the same changes, and there were statistical differences among groups, *P<0.05.


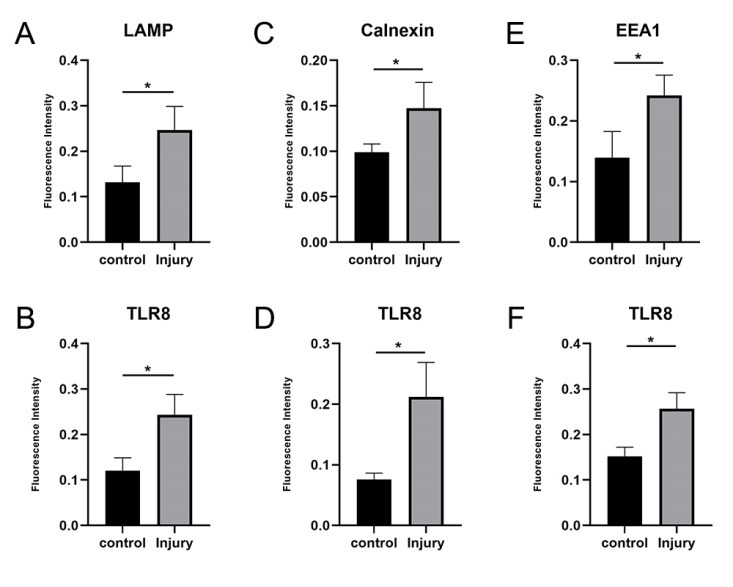


**Fig.S9:** Quantitative analysis for LAMP, Calnexin and EEA1 immunfluorescent results,*P<0.05.
